# Correlation of Functional and Structural Outcomes with Serum Antibody Profiles in Patients with Neovascular Age-Related Macular Degeneration Treated with Ranibizumab and Healthy Subjects: A Prospective, Controlled Monocenter Trial

**DOI:** 10.3390/jcm13237033

**Published:** 2024-11-21

**Authors:** Christina A. Korb, Eva Gerstenberger, Katrin Lorenz, Katharina Bell, Anna Beck, Yvonne Scheller, Vanessa M. Beutgen, Dominik Wolters, Franz H. Grus

**Affiliations:** 1 Department of Ophthalmology, University Medical Center, Johannes Gutenberg-University, Langenbeckstrasse 1, 55131 Mainz, Germany; 2 NHMRC Clinical Trials Centre, University of Sydney, Camperdown, NSW 2050, Australia; 3 Interdisciplinary Center Clinical Trials Mainz, University Medical Center, Johannes Gutenberg-University, 55131 Mainz, Germany

**Keywords:** age-related macular degeneration, neovascular, antibodies, ranibizumab

## Abstract

**Background:** Age-related macular degeneration (AMD) is a multifactorial disorder, and there is growing evidence of immunological involvement in its pathogenesis. To address this, we aimed to identify biomarker candidates related to retinal antigens in patients with neovascular AMD treated with ranibizumab and healthy subjects. **Materials and Methods:** This study was designed as a prospective, open, parallel-group, interventional, single-center phase IV trial. Fifty subjects with neovascular AMD and twenty healthy volunteers were enrolled. The primary objective was to assess the efficacy of intravitreally (IVT) administered ranibizumab in terms of the change in best-corrected visual acuity in subjects with all subtypes of neovascular AMD and in a subgroup of pretreated AMD subjects. A secondary objective was to assess the efficacy of the same in terms of the change in central retinal thickness (CRT) in the same subjects. Another secondary objective was to identify antibodies against retinal antigens in patients with neovascular AMD treated with ranibizumab and healthy subjects. The last secondary objective was to correlate functional and structural parameters with the identified biomarker candidates to differentiate between initial and deferred responders to IVT administered ranibizumab. Serum was analyzed using customized antigen microarrays containing 58 antigens. **Results:** After 12 weeks of ranibizumab treatment, treated patients gained 4.02 letters on average. The central retinal thickness (CRT) measured in the complete AMD study population was significantly (*p* < 0.001) decreased at Week 24 compared to the baseline measurement, and the mean CRT dropped from 393.4 to 296.8 µm. A significant increase in the following autoantibodies was detected between the control group and AMD group at Week 24, as well as in the AMD group between baseline and Week 24: antibodies targeting the proteins serotransferrin, opioid growth factor receptor, 60 kDa chaperonin 2, neurotrophin-4, dermcidin, clusterin and vascular endothelial growth factor. **Conclusions:** The present trial was able to confirm the efficacy of ranibizumab treatment in neovascular AMD, and treatment-naïve patients benefitted the most. Up- and downregulations of antibodies were observed over the course of treatment with ranibizumab. Some antibodies seemed to have a fair correlation with the classification of initial and deferred responders.

## 1. Background

Age-related macular degeneration (AMD) is a multifactorial disorder and a significant factor leading to permanent vision impairment and severe vision loss in developed countries [1]. Anti-retinal autoantibodies play a significant role in various ocular conditions including retinal vasculitis [2], paraneoplastic and autoimmune retinopathy [3,4,5], retinitis pigmentosa [6], myopic macular degeneration [7] and age-related macular degeneration (AMD) [8,9,10,11,12,13,14], and there is an increasing amount of evidence pointing towards the involvement of the immune system in the development of AMD. In accordance, we have previously shown a notable distinction in the immunoglobulin G (IgG) antibody profiles against retinal antigens, with patients diagnosed with wet AMD exhibiting different patterns compared to healthy volunteers. Accordingly, we previously demonstrated a significant difference in the patterns of immunoglobulin G (IgG) antibodies against retinal antigens between patients with wet AMD and healthy volunteers [15]. Furthermore, a recent comparison of autoantibody profiles in patients with dry and wet AMD revealed significantly altered immunoreactivities against proteins particularly found in immunological diseases, in addition to neurodegenerative, apoptotic and autoimmune markers [16].

We cannot currently answer the question of whether these antibody changes appear prior to AMD onset or rather after developing the disease. In this prospective, controlled, monocenter trial study, we therefore aimed to explore the following aspects in much more detail: the efficacy of intravitreal (IVT) ranibizumab (the changes in best-corrected visual acuity (BCVA) and central retinal thickness (CRT)) in subjects with all subtypes of neovascular AMD and in a subgroup of pretreated AMD subjects. Furthermore, in this study, we aimed to identify biomarker candidates related to retinal antigens in patients with neovascular AMD treated with ranibizumab and healthy subjects. Lastly, we aimed to correlate functional and structural parameters (BCVA and central retinal thickness, CRT) with the identified biomarker candidates to differentiate between initial and deferred responders to IVT administered ranibizumab.

## 2. Methods

### 2.1. Trial Design

This study was designed as a prospective, open, parallel-group, interventional, single-center phase IV trial performed at the Department of Ophthalmology, University Medical Center, Johannes Gutenberg-University Mainz, Germany. The study was conducted according to the guidelines of the Declaration of Helsinki and approved by the Ethics Committee of Landesärztekammer Rheinland-Pfalz (18 May 2016; handling code 837.150.16(10469)) and by the competent authority Paul-Ehrlich-Institut (19 July 2016; handling code 2751/01) before the study was started. The clinical trial was registered at ClinicalTrials.gov (identifier: NCT 02843490) before the first patient was screened. Prior to their inclusion, every patient provided informed consent. According to the sample size calculations, fifty patients and twenty healthy volunteers were required.

The duration of the trial per patient was 6 months with monthly blood collections; healthy volunteers were enrolled only for one blood collection. The initial treatment of monthly ranibizumab injections (Lucentis^®^, Novartis Pharma GmbH, Nürnberg, Germany) was provided during the first three months, followed by an individual therapy interval based on the clinical progress (pro re nata, PRN).

A list of the inclusion and exclusion criteria and an overview of the trial schedule are available in the Appendix A.

### 2.2. Objectives

The primary objective was to assess the efficacy of intravitreally (IVT) administered ranibizumab in terms of the change in best-corrected visual acuity (BCVA) in subjects with all subtypes of neovascular AMD and in a subgroup of pretreated AMD subjects. A secondary objective was to assess the efficacy of the same in terms of the change in central retinal thickness (CRT), as measured through optical coherence tomography (OCT) (Spectralis OCT, Heidelberg Engineering, Heidelberg, Germany), in the same subjects. Another secondary objective was to identify antibodies (biomarker candidates) against retinal antigens in patients with neovascular AMD treated with ranibizumab and healthy subjects, as well as to validate the same found in previous studies. The last secondary objective was to correlate functional and structural parameters (BCVA and CRT) with the identified biomarker candidates to differentiate between initial and deferred responders to IVT administered ranibizumab.

### 2.3. Patients

A total of 70 subjects aged 50 and over were enrolled in the clinical trial, i.e., 50 subjects with neovascular AMD (plus 1 screening failure) and 20 healthy volunteers. The statistical calculations of sample sizes were based on experiences from previous studies with similar methodologies and questioning (e.g., [16,17]). The recruitment and treatment of subjects were performed at the clinical trial center of the Department of Ophthalmology, University Medical Center, Johannes Gutenberg-University Mainz, Germany.

### 2.4. Microarray Antibodies

To screen autoantibody reactivities, we used an advanced high-density microarray approach. Customized microarrays were manufactured in our lab with the help of a noncontact piezo-dispenser (SciFLEXARRAYER S3, Scienion, Berlin, Germany). The 58 chosen antigens were spotted in triplicate onto nitrocellulose-coated microarray slides (AVID Oncyte, 16 Pad NC slides, Grace Biolabs, Bend, OR, USA), along with human IgG-Mix (Sigma-Aldrich Chemie GmbH, Taufkirchen, Germany) as a positive control spot and spotting buffer (PBS) as a negative control spot. A list of selected antigens is available in the Appendix A. The spotting process took place in a humidity chamber, with the humidity level set at 60%. For the proteins on the microarray surface to be immobilized optimally, the slides were left on the spotter platform to dry overnight before incubation. During the incubation steps, the slides were placed in incubation chambers (ProPlate Multiwell chambers, Grace Biolabs, Bend, OR, USA), effectively dividing each slide into 16 separate subarrays. The subsequent incubation steps were carried out at a temperature of 4 °C, using an orbital shaker. To minimize background signals, the arrays were incubated with blocking buffer (Super G, Grace Biolabs, Bend, OR, USA) for one hour. After that, the blocking buffer was removed, and any remaining buffer was removed by washing three times with phosphate-buffered saline containing 0.5% Tween-20 (PBST, ICN Biomedicals, Meckenheim, Germany). The arrays then were incubated with 100 µL serum samples in a 1:250 dilution in PBS overnight. One subarray on each slide served as a negative control and was incubated with PBS rather than serum. The slides underwent an additional three washes with PBST and were then incubated for one hour with a secondary anti-human IgG antibody conjugated with Alexa fluor 647 (Alexa Fluor^®^ 647 AffiniPure Goat Anti-Human IgG, Fcγ fragment specific, 109-605-008, Jackson Immunoresearch, Ely, Cambridgeshire, UK) in a 1:500 dilution in PBS. After incubating with the secondary antibody, the slides were washed two times with PBST and two times with ultrapure water. The slides were then dried for two minutes in a vacuum concentrator (SpeedVac, Thermo Scientific, Waltham, MA, USA).

The microarray slides were scanned with a high-resolution confocal laser scanner (428 Array Scanner, Affymetrix, Santa Clara, CA, USA). The scans were stored as 16-bit TIFF images. The spot intensities were measured with image-processing software (Imagene 5.5, BioDiscovery Inc., Los Angeles, CA, USA). Spots with poor quality were manually identified and excluded from further analysis.

To correct for the unspecific binding of the secondary antibody, the intensities of the negative control on each slide were subtracted from the spot intensities of the samples. The median fluorescence intensities of the three replicate spots were averaged, yielding one expression value for each antigen. To account for technical variance in the array fabrication process, the data were normalized to the median-centered values of the positive control spots on each subarray. All the statistical analyses were conducted on the normalized fluorescence intensities (NFIs).

### 2.5. Study Endpoints and Statistical Analysis

The primary endpoint was the change in BCVA score from baseline (Visit 1 = V1) to Week 12 (Visit 4) in the study eye. The secondary efficacy endpoints included the change in BCVA score from baseline (V1) to Week 24 (V7) in the study eye, as well as the absolute change in central retinal thickness from baseline (V1) to Week 24 (V7), as assessed by OCT. Another secondary endpoint was the mean number of IVT ranibizumab injections needed up to Week 24 (V7) in the study eye.

The visual function of both the study and fellow eyes was assessed using ETDRS charts [18]. The baseline points were compared with endpoints regarding different parameters (the retinal thickness, the mean number of IVT injections, the antibody autoreactivity values and further clinical parameters) using paired two-sided *t*-tests.

The patient group was divided into two subgroups: AMD patients with newly diagnosed, treatment-naïve neovascular AMD and pretreated AMD patients with neovascular AMD who had not received any anti-VEGF treatment 3 months prior to inclusion in the study. The analysis of the subgroup of pretreated AMD subjects was included in the primary efficacy analysis strategy. As an exploratory analysis, the subgroup of naïve neovascular AMD patients was also analyzed.

The study population was divided into subjects who had lost no letters in the ETDRS letter score by Week 12 in comparison to Visit 1 (“initial responders”) and subjects who had lost at least one letter by that point (“deferred responders”). The correlation of functional and structural parameters (BCVA and CRT) with the identified biomarkers was also analyzed to differentiate between initial and deferred responders. To assess the group differences in the tested autoantibodies between the control group, the AMD group at timepoint V1 and the AMD group at V7, a nonparametric test was applied. The Kruskal–Wallis ANOVA revealed significant changes in the levels of autoantibodies to some of the antigens tested in this setup.

Using a multivariate binomial logistic regression analysis, the possible correlates, clinical parameters and antibody reactivities of the medication responder type were analyzed, and it was determined whether these variables could predict the responder type of an AMD patient.

The statistical analysis was performed in TIBCO Statistica (v. 13.3.0, TIBCO Software Inc., Palo Alto, CA, USA) and in R version 3.3.1 (R Core Team (2016); R: A language and environment for statistical computing; R Foundation for Statistical Computing, Vienna, Austria, with the following URL: https://www.R-project.org/ (accessed on 1 August 2016).

## 3. Results

The patients in the AMD group were 78.48 (±8.51) years old on average, and those in the control group were 71.05 (±9.37) years old on average. The control group comprised 15 (75%) females and five (25%) males. There were 26 (52%) females and 24 (48%) males in the AMD group (Table 1).

The study population was categorized into two groups based on their treatment history and response status. Out of the 49 participants, 38 (77%) were treatment-naïve, having never undergone prior therapy, while the remaining 11 (33%) participants had previously received treatment.

Based on the Week 12 ETDRS score, 33 (67.35%) individuals were identified as initial responders, demonstrating a prompt response to the treatment. The other 16 (32.65%) participants were classified as deferred responders.

One patient in the AMD group was not included because they discontinued with the study before their responder type could be assessed.

### 3.1. Changes in ETDRS Score

**Figure 1:**
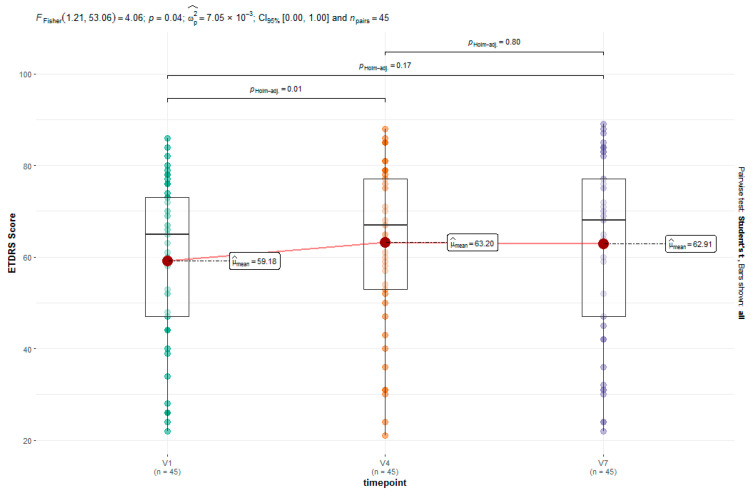
Changes in ETDRS score for primary endpoint (V1 vs. V4) and secondary endpoint (V1 vs. V7).

Regarding the ETDRS scores of all the AMD patients, a significant increase (*p* = 0.01) in the BCVA could be observed at Week 12 (V4). At baseline, the mean ETDRS letter score of the study population was at 59.18. After 12 weeks of ranibizumab treatment, this increased to 63.20. The treated patients gained 4.02 letters on average (Figure 1).

In the subgroup of treatment-naïve patients, the ETDRS score increased significantly by 4.23 letters on average, with a significant increase in BCVA score (*p* = 0.001). In the group of pretreated AMD subjects (patients who had not undergone any anti-VEGF treatment for three months before being included in the study but had received ranibizumab injections before), the mean ETDRS score increased from 66.4 to 69.7. This average increase of 3.3 letters failed to prove a significant effect.

**Figure 2:**
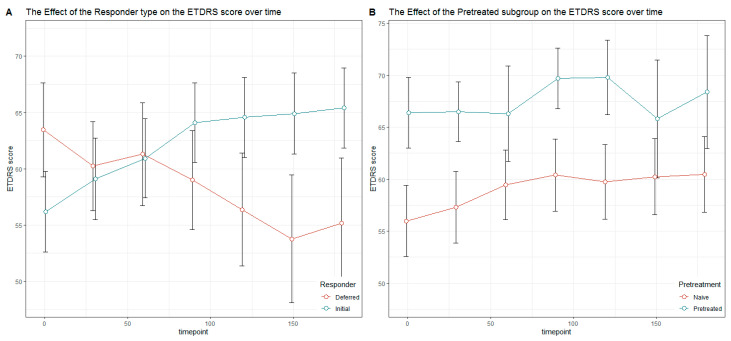
Courses of ETDRS scores over all the assessment times in the group of initial and deferred responders (**A**) as well as treatment-naïve and pretreated patients (**B**).

Furthermore, the changes in BCVA were evaluated over the complete study duration. The mean ETDRS letter score of the AMD patients treated with ranibizumab increased from 59.18 to 62.91, but this effect did not meet the 0.05 level for significance. Regarding only treatment-naïve AMD patients without prior treatment, an average increase of 4.2 in the ETDRS score was observed, but the effect was likewise not significant. Moreover, there was no significant change in BCVA in the subgroup of pretreated AMD patients (Figure 2). The courses of the ETDRS scores over all the assessment times in the group of initial and deferred responders as well as treatment-naïve and pretreated patients are also shown in Figure 2.

### 3.2. Changes in Central Retinal Thickness

**Figure 3:**
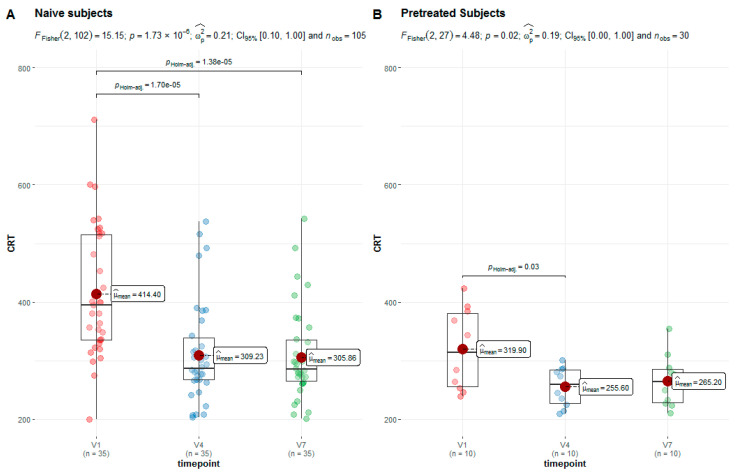
CRT values (µm) in the groups of treatment-naïve (**A**) and pretreated (**B**) patients for the primary timepoints.

The changes in central retinal thickness (CRT) in the study population were assessed through OCT. Compared to the baseline measurement, the CRT measured in the complete AMD study population was significantly (*p* < 0.001) decreased at Week 24 (V7). The mean CRT decreased from 393.4 to 296.8 µm. The CRT in the subgroup of naïve AMD patients significantly (*p* < 0.001) decreased from a mean of 414.4 µm at baseline to a mean of 305.9 µm at Week 24. The subgroup of pretreated AMD patients also showed a significant (*p* = 0.03) decrease in CRT at timepoint V4 compared to V1. The mean CRT decreased from 319.9 to 255.6 µm on average. The pretreated patients initially had a thinner mean CRT compared to treatment-naïve patients (Figure 3).

**Figure 4:**
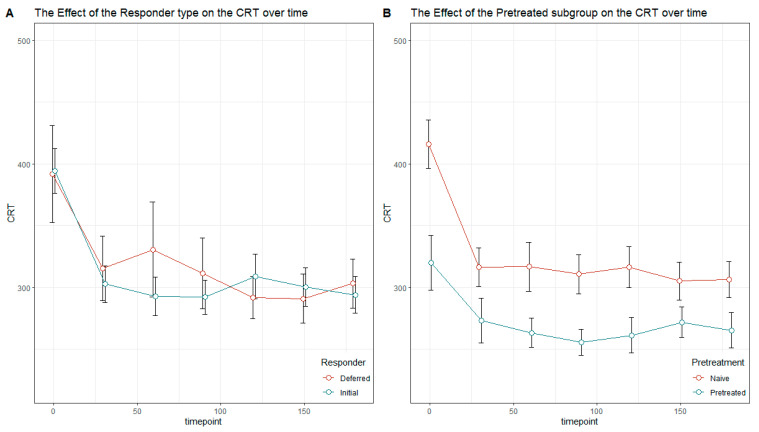
Courses of CRT (µm) over all the assessment times in the deferred versus initial responder group (**A**) and the group of treatment-naïve compared to pretreated patients (**B**).

The courses of the central retinal thickness over all the assessment times in the deferred versus initial responder group and the group of treatment-naïve compared to pretreated patients is shown in Figure 4.

### 3.3. Number of Ranibizumab Injections

The mean number of IVT ranibizumab injections in the AMD group was 4.75. The mean number of injections showed no significant alterations in all the AMD subgroups of deferred and initial responders and treatment-naïve or pretreated patients.

### 3.4. Exploratory Analysis Results

Several significant changes in serological autoantibody levels were identified upon comparing the control group and the AMD group, as well as timepoints V1 and V7 in the AMD group (Table 2).

Anti-transthyretin (TTR) levels were significantly decreased at timepoint V7 in the AMD group compared to the control group. Antibodies against carbonic anhydrase 2 (CA2), aconitate hydratase (ACO2), 60 kDa heat shock protein (HSPD1), myelin basic protein (MBP), superoxide dismutase (SOD), gamma-synuclein (SNCG) and serum albumin (ALB) were significantly increased when comparing these two groups.

A significant increase in autoantibody levels from V1 to V7 in the AMD group was observed for mucin, brain-derived neurotrophic factor (BDNF), calreticulin (CALR) and neurotrophin-3 (NTF3).

**Figure 5:**
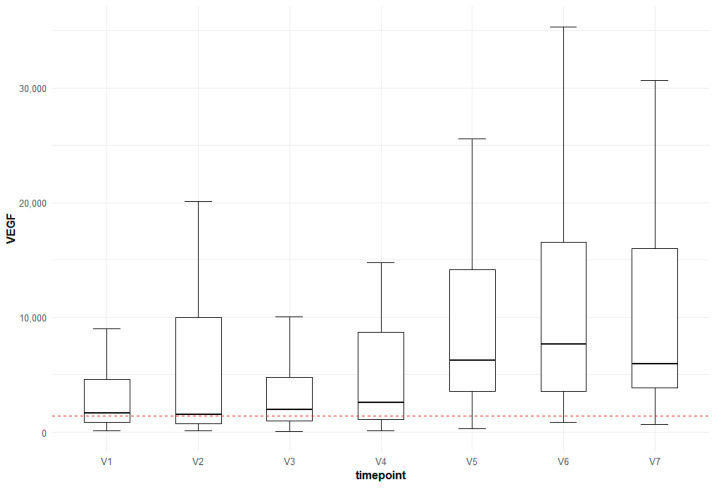
Box-plot for the change in anti-VEGF levels over time compared to the control group (red horizontal line).

**Figure 6:**
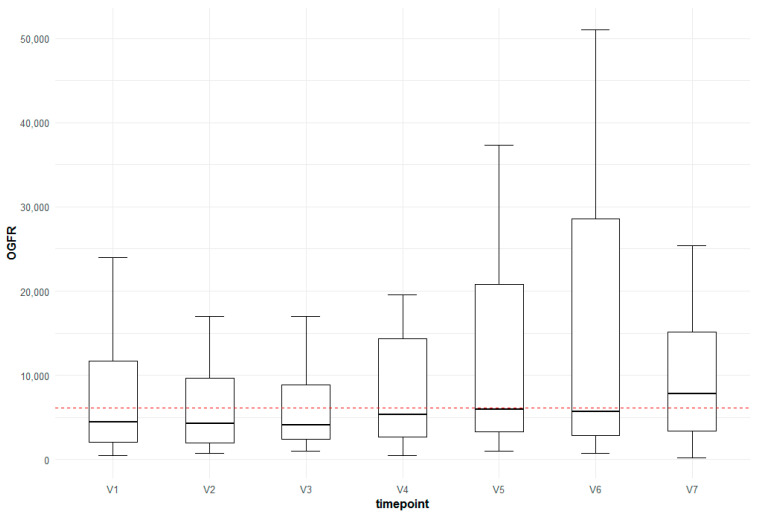
Box-plot for the change in anti-OGFR levels over time compared to the control group (red horizontal line).

For the following autoantibodies, significant increases between the control group (CTRL) and AMD V7, as well as between AMD V1 and V7, were detected: antibodies targeting the proteins serotransferrin (TF), opioid growth factor receptor (OGFR), 60 kDa chaperonin 2 (groEL2), neurotrophin-4 (NTF4), dermcidin, clusterin (CLUS) and vascular endothelial growth factor (VEGF) (Figure 5 and Figure 6).

Overall, there was no significant change in autoantibody levels between the CTRL and AMD V1s. Each of the identified biomarkers was then correlated with functional and structural parameters to differentiate between initial and deferred responders.

Correlation analysis suggests only weak correlation of each of the tested variables with the medical responder type (Table 3). The logistic regression model shows that the refraction parameters (spherical and cylinder), as well as the antibody levels for gamma-synuclein (SNCG), eukaryotic initiation factor 4A-I (EIF4A1) and glucosidase 2 subunit beta (PRKCSH), are significant predictors of the responder type. The influence of the antibody variables on the probability of being a responder, however, is marginal, as can be seen from the low odds ratios (Table 3).

**Figure 7:**
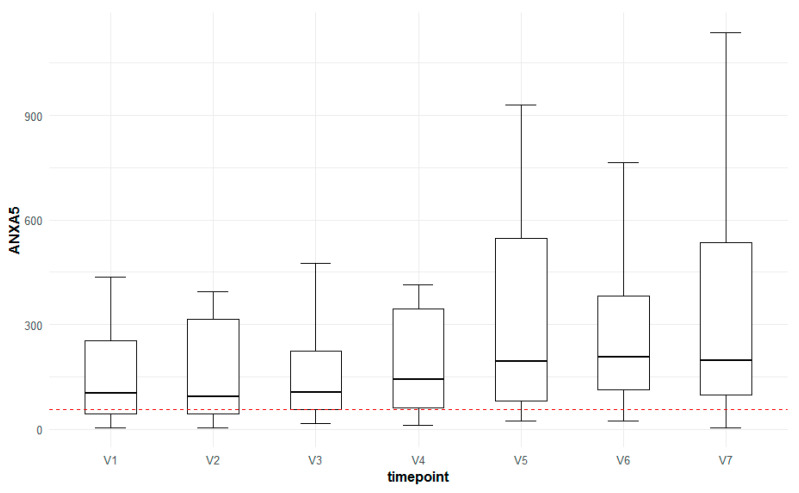
Box-plot for the change in anti-annexin A5 levels over time compared to the control group (red horizontal line).

Regarding the courses of the autoantibody levels in the AMD patients treated with ranibizumab, seven showed significant changes at some point during the study. These were antibodies to annexin A5 (ANXA5) (Figure 7), superoxide dismutase (SOD), gamma-synuclein (SNCG), insulin (INS), beta L-crystalline (B-L-CRYS), thyroglobulin (TG) and aconitase 2 (ACO2). However, it cannot be concluded whether any of these differences were time- or treatment-dependent because no longitudinal data were collected in the control group.

### 3.5. Safety and Adverse Events (AEs)

Regarding the safety aspects, no serious adverse events related to the treatment were reported. A total of 18 AEs related to the injection procedure were documented, but none of them were judged as serious.

## 4. Discussion

In the present study, the increment in BCVA mainly occurred during the first three months, with scheduled ranibizumab injections. Upon starting the PRN treatment, the vision of the AMD patients remained constant. The strongest effects on the increment in the ETDRS letter score occurred during the first 3 to 4 months of treatment with ranibizumab, in accordance with observations made in the pivotal MARINA [19] and ANCHOR [20] studies.

However, treatment-naïve patients starting ranibizumab for the first time benefitted the most from treatment. In this group, a significant increase in the ETDRS letter score from baseline to Week 12 (+4.1) could be observed; however, there was no further increase in VA by Week 24 (+4.2). These data suggest that the improvement in BCVA is most prominent at the beginning of the treatment, and vision can be sustained in the long term. The mean improvement in BCVA observed in the CAPTAIN study, however, does not match the ETDRS letter gains observed after 12 months with 0.5 mg ranibizumab doses in the MARINA (+7.2) [19] or ANCHOR study (+11.3) [20]. However, the mean changes in BCVA in these studies were assessed after 12 months with monthly scheduled ranibizumab injections.

Regarding only the pretreated individuals who had received ranibizumab in the past, there was no significant change in BCVA during the 24 weeks of this study. On the other hand, the subgroup analysis revealed distinct differences between the treatment-naïve and pretreated patients (baseline mean ETDRS score of treatment-naïve patients: 58 letters; baseline mean ETDRS score of pretreated patients: 62 letters). These observations are described as the so-called “ceiling effect”, whereby, in patients with better initial visual capacity receiving anti-VEGF injections over time, the maximum visual gain attainable is limited [21]. As the ceiling effect can limit improvements in eyes with relatively good baseline vision, this phenomenon may skew the results.

The mean injection frequency was 4.75 during the six-month duration of our study. A systematic literature review revealed a mean improvement in visual acuity gain of 5.4 ETDRS letters in the first year with a mean of 5.6 intravitreal injections of ranibizumab or bevacizumab administered with a PRN regimen [22]. In general, it is considered that the approximate required number of injections for neovascular AMD with PRN treatment is seven per year [23,24], although sustained higher gains are possible with more intense regimens, underlining the importance of avoiding undertreatment [25].

As a secondary objective, the efficacy of intravitreal ranibizumab injections in terms of the change in the central retinal thickness was assessed. Regardless of the subgrouping, the CRT decreased significantly from baseline to Visit 2, one month after the first ranibizumab injection. After V2, no significant changes occurred, and the CRT stabilized around an average of 300 µm. The pretreated patient population showed less CRT reduction in comparison to the naïve patient population; however, the mean baseline CRT was significantly thinner in the group of pretreated patients. These data underline the immediate effect of the intravitreal injection on the central retinal thickness. A variable dosing regimen in the PrONTO study [26] also led to a significant decrease in retinal thickness, which continued through the first three intravitreal injections and was maintained for 24 months [27].

The exploratory analysis of autoantibody levels revealed no significant changes in autoantibody levels for the tested targets between the control group and the group of patients with neovascular AMD at baseline. Nevertheless, significantly increased and decreased levels in some autoantibodies were detected between the CTRL and AMD groups at Visit 7. Several autoantibodies showed significantly increased serological levels between the control group and AMD group at V7, as well as between V1 and V7 in the AMD group. These antibodies target the proteins serotransferrin (TF), opioid growth factor receptor (OGFR), 60 kDa chaperonin 2 (groEL2), neurotrophin 4 (NTF4), dermcidin (DCD), clusterin (CLUS) and vascular endothelial growth factor (VEGF). Transferrin may function as a component of the retinal oxidative defense system and showed an increase in the retinas of patients with AMD in contrast to healthy controls [28]. Wysokinski et al. also showed that the level of transferrin in the serum was elevated in the AMD group as opposed to the control group and described genetic polymorphisms in iron homeostasis genes as likely risk markers for AMD [29]. In our study, autoantibodies against serotransferrin were significantly increased in the AMD group between Visits 1 and 7.

Ranibizumab is an anti-VEGF-A affinity-matured monovalent monoclonal 48 kDA antibody fragment [30,31]. It does not contain an FC antibody region and is therefore subject to systemic catabolism in the systemic circulation [32]. Gu et al. found no significant changes in serum VEGF levels from days 3 to 30 following a single intravitreal ranibizumab injection [33]. On the other hand, therapeutic biologic agents targeting vascular endothelial growth factors can initiate immune responses and thus produce antidrug antibodies (ADAs) [34,35]. Bressler et al. recently suggested that immunogenicity was not associated with the efficacy and safety of ranibizumab products [34]. Lee et al. did not detect ADAs in the aqueous humor of patients with ranibiziumab-recalcitrant neovascular AMD and concluded that ADAs do not directly interfere with the action of intraocular ranibizumab [35]. Systemic immunoreactivity to ranibizumab has been reported previously and seems to increase over time, although the clinical significance remains unclear [19,20,35,36]. Mettu and coworkers stated that the development of neutralizing antidrug antibodies directed against anti-VEGF biologics is a potential cause of the loss of drug effectiveness [37]. In the Marina Phase 3 ranibizumab study, by the end of the second year of intravitreal treatment, the immunoreactivity rates were 4.4% in the 0.3 mg group and 6.3% in the 0.5 mg group, as compared with only 1.1% in the sham-injection group [19]. Accordingly, in the present study, in particular, there were significant changes in the VEGF antibody levels between the control group and treated patients as well as between untreated and treated patients (AMD 1 and 7), highlighting a possible treatment-induced effect.

In a former study, we reported that autoantibodies against the opioid growth factor receptor (OGFR) were differently regulated in patients with dry and neovascular AMD compared to controls [16]. In the present study, autoantibodies against OGFR showed a significant increase over time compared to the control group. The antibody levels were significantly altered in the control group versus AMD 7 group as well as in the AMD 1 versus AMD 7 group. This underlines possible treatment-related effects. The opioid growth factor ([Met5]-enkephalin) is an endogenous pentapeptide that binds to OGFR. This regulatory pathway plays a crucial role for homeostasis in cell replication and renewal and has receptor-mediated action in regulating angiogenesis [38,39]. Opioid antagonists like naloxone are effective treatments for cancer, autoimmune disorders and diabetes-related complications [40]. Furthermore, in mice, naloxone significantly slowed down the advancement of retinal AMD-like lesions by regulating the accumulation and activation of microglia at the site of retinal degeneration [41]. Husain et al. showed that opioid receptor activation plays a central role in the suppression of TNF-α and might protect retinal function [42]. In the present study, autoantibodies against OGFR showed a significant increase over time compared to the control group and also between timepoints 1 and 7 in the AMD group, suggesting a possible therapy-induced effect. These observations suggest that the OGF–OGFr axis might also play a critical part during anti-VEGF treatment, and the role of these autoantibodies warrants verification and further investigation.

Regarding the courses of the autoantibody levels in the AMD patients treated with ranibizumab, seven antibodies showed significant changes at some point during the study. These were antibodies to annexin A5 (ANXA5), superoxide dismutase (SOD), gamma-synuclein (SNCG), insulin (INS), beta L-crystalline (B-L-CRYS), thyroglobulin (TG) and aconitase 2 (ACO2). However, it cannot be concluded whether any of these differences were time- or treatment-dependent because there were no longitudinal data for the control group, with only one visit at baseline.

In a previous study, we were able to show that immunoreactivities against annexin A5 were higher in patients with AMD compared to control [16]. Annexin A5 is a protein that binds to phospholipids, playing a significant role in regulating apoptosis. It is highly expressed by vascular endothelial cells [43]. Moreover, cytosolic annexin A5 plays a crucial role in the particle-binding step of clearance phagocytosis, an essential process in retinal physiology [44]. Increased levels of annexin A5 mRNA transcripts were found in peripheral white blood cells, and it was suggested that levels of annexin A5 may rise in AMD as part of a healing response [45]. Additionally, anti-annexin A5 was previously found to be upregulated in the serum of AMD patients. It is believed to play a role in AMD pathogenesis through an autophagy-mediated mechanism [14]. It was recently shown that annexin A1 inhibited angiogenic and pro-inflammatory cytokines and promoted reparative angiogenesis; thus, a neuronal protective function in ischemic retinopathy was described [46].

Previous studies indicated a potential contribution of the synuclein family to retinal neurodegeneration, especially in the context of remodeling [47,48]. Liu et al. suggested that gamma-synuclein plays a crucial role in the apoptosis of retinal ganglion cells and the degeneration of the optic nerve [49]. A recent study demonstrated that the synuclein distribution in the mouse retina is altered with aging [50]. In the present study, the different immunoreactivities against annexin A5 and gamma-synuclein during the course of the intravitreal therapy might suggest that these autoantibody levels could change over the course of the intravitreal therapy or disease progression. Furthermore, the analysis of antibody titers in a larger patient cohort with neovascular AMD might be valuable, especially if the inactivation or removal of specific antibodies could be useful in monitoring or have a therapeutic effect in treating neovascular AMD.

The logistic regression analysis showed that spherical and cylindrical refraction, as well as the levels of autoantibodies to gamma-synuclein (SNCG), eukaryotic initiation factor 4A-I (EIF4A1) and glucosidase 2 subunit beta (PRKCSH), were significant predictors of the responder type. The whole model shows an AURROC of 0.78 for the classification of initial and deferred responders, which indicates fair classification power. An incomplete response to intravitreal therapy, especially persistent disease activity and suboptimal vision recovery, represents significant clinical unmet needs [37]. Most of the intravitreally treated patients with neovascular AMD experience stable or improved vision, although some patients lose visual acuity [51]. The influence of the antibody variables on the probability of being a responder, however, was only marginal.

Some of this study’s shortcomings should be openly underlined. The study encountered challenges with unequal demographics between the control group and the AMD group, as well as among the AMD subgroups, potentially introducing bias and impacting the outcomes. There were unequal group sizes among the treatment-naïve and pretreated patients, initial and deferred responders, and control and AMD group as a whole, complicating the interpretation of the results and limiting the statistical power of the analysis. Furthermore, the AMD group and control group were neither age- nor gender-matched, which could have led to confounding variables influencing the outcomes. The absence of proper matching could have impacted the reliability of the conclusions drawn from the comparisons. Lastly, the study duration of 24 weeks was relatively short, limiting our ability to assess the long-term effects and outcomes of the treatment. Extended follow-up periods could enable a more thorough understanding of the effectiveness and safety of ranibizumab.

The strengths of the present study are its prospective nature and the analysis using customized antigen microarrays containing 58 selected antigens. Antigen microarrays allow for the fast and highly sensitive evaluation of potential autoantibody biomarkers using small microliter sample volumes. Spectral-domain OCT imaging was used in all patients. Functional and structural parameters were correlated with the identified biomarkers to differentiate between initial and deferred responders to IVT administered ranibizumab to facilitate the future improvement of patients’ disease treatments and outcomes, possibly through individual precision medicine.

Despite some flaws caused by the unequal subgroup sizes, the present trial was able to confirm the efficacy of ranibizumab treatment in neovascular AMD. Treatment-naïve patients benefitted most significantly.

Furthermore, down- and upregulations of antibodies were observed over the course of treatment with ranibizumab. Some antibodies seemed to have a fair correlation with the classification of initial and deferred responders; thus, further investigation is needed and will be useful in the field of ocular immunology.

## Figures and Tables

**Table 1:** Demographics of study population.

Variable	CTRL	AMD	Total
N	20	49	69
Age [years]; mean (SD)	71.05 (9.37)	78.48 (8.51)	76.36 (9.33)
Female	15 (75.00%)	26 (52.0%)	41 (58.57%)
Male	5 (25.00%)	24 (48.0%)	29 (41.43%)
Treatment-naïve		38 (77.0%)	38 (77.0%)
Pretreated		11 (33.0%)	11 (33.0%)
Responder: initial		33 (67.35%)	33 (67.35%)
Responder: deferred		16 (32.65%)	16 (32.65%)

**Table 2:** Results of the autoantibody reactivities (mean ± standard deviation) along with the corresponding *p*-values obtained from a Kruskal–Wallis ANOVA followed by Dunn’s post-hoc test. *p* values of significantly changed autoantibody levels using Benjamini–Hochberg procedure are marked with * (FDR < 0.1) and ** (FDR < 0.05).

	CTRL (n = 20)	AMD V1 (n = 51)	AMD V7 (n = 45)	CTRL vs. AMD V1	CTRL vs. AMD V7	AMD V1 vs. AMD V7
NTF3	56,118 ± 148,412	25,089 ± 29,299	60,782 ± 81,897	1.000	0.25	0.011 **
ACO2	415 ± 940	429 ± 525	706 ± 975	0.769	0.043 *	0.241
HSPD1	28,166 ± 28,430	51,236 ± 81,773	90,819 ± 144,239	0.753	0.034 *	0.195
ALB	6169 ± 12,010	11,504 ± 19,920	21,661 ± 55,524	0.967	0.048	0.178
TTR	1566 ± 6470	325 ± 429	590 ± 841	0.216	0.004 **	0.177
CA2	82,278 ± 137,370	82,870 ± 86,933	131,123 ± 137,132	1.000	0.042 *	0.067
MBP	5302 ± 15,824	4448 ± 5340	11,875 ± 28,819	0.24	0.001 **	0.061
SNCG	6844 ± 8510	11,655 ± 21,215	15,918 ± 30,304	1.000	0.043 *	0.051
groEL2	25,625 ± 28,341	50,827 ± 83,165	78,762 ± 117,962	0.655	0.005 **	0.032 *
CALR	65,456 ± 127,619	41,690 ± 45,664	88,560 ± 128,165	0.881	0.998	0.026 *
Mucin 5B	4438 ± 11,390	2477 ± 2527	5710 ± 7395	1.000	0.079	0.019 *
SOD	775 ± 1966	1409 ± 2695	1950 ± 4315	0.353	0.015 *	0.277
NTF4	3070 ± 6568	2294 ± 3202	4858 ± 6272	1.000	0.028 *	0.007 **
OGFR	6354 ± 11,293	4724 ± 6154	13,931 ± 38,235	1.000	0.006 **	0.005 **
BDNF	27,360 ± 36,641	20,753 ± 35,313	38,897 ± 86,989	0.351	1.000	0.005 **
Dermcidin	10,111 ± 14,190	13,797 ± 34,140	19,041 ± 20,508	1.000	0.045	0.003 **
CLUS	4696 ± 10,040	3672 ± 4518	9231 ± 11,712	1.000	0.006 **	0.003 **
TF	16,309 ± 29,591	13,116 ± 23,293	32,492 ± 55,885	1.000	0.036 *	0.002 **
VEGF	7918 ± 22,695	5423 ± 10,712	13,302 ± 17,109	1.000	0.001 **	<0.001 **

**Table 3:** Logistic regression analysis (initial = 1; deferred = 0).

							OR 95% CI	
	Estimate	SE	Wald	df	*p* Value	Odds Ratio	Lower	Upper
Intercept	1.556	1.740	0.800	1	0.371			
Refraction spherical	0.259	0.109	5.613	1	0.018	1.296	1.046	1.606
Refraction cylinder	0.802	0.364	4.848	1	0.028	2.229	1.092	4.549
Refraction degree	0.001	0.007	0.050	1	0.823	1.001	0.989	1.015
Central retinal thickness	0.000	0.001	0.163	1	0.686	1.000	0.997	1.002
ETDRS score	0.010	0.014	0.507	1	0.476	1.010	0.983	1.038
No. of IVT injections	−0.143	0.195	0.542	1	0.462	0.867	0.592	1.269
APOA1	0.000	0.000	0.546	1	0.460	1.000	1.000	1.000
USP10	0.000	0.000	3.092	1	0.079	1.000	1.000	1.000
SFN	0.000	0.000	3.600	1	0.058	1.000	1.000	1.000
SNCG	0.000	0.000	4.194	1	0.041	1.000	1.000	1.000
EIF4A1	0.001	0.000	11.313	1	0.001	1.001	1.000	1.001
PRKCSH	0.000	0.000	15.042	1	<0.001	1.000	1.000	1.000
GPX4	0.000	0.000	0.319	1	0.572	1.000	1.000	1.000

## Data Availability

The original contributions presented in the study are included in the article, further inquiries can be directed to the corresponding authors.

## Appendix A. Enrollment Criteria

List of inclusion and exclusion criteria.

*Inclusion criteria*

**Patients:**

Subjects meeting all of the following criteria, which is also the criteria for the diagnosis of neovascular AMD in the study eye, will be considered for admission to the trial:

- Male or female;
- Age ≥ 50 years;
- Subfoveal, juxtafoveal and/or extrafoveal choroidal neovascularisation due to neovascular age-related macular degeneration in the study eye;
- Visual acuity of 20/400 (ETDRS charts) or better in the study eye;
- Ability of subject to understand character and individual consequences of the clinical trial.

Signed and dated informed consent of the subject must be available before the start of any specific trial procedures.

Women with childbearing potential have to practice a medically accepted contraception during the trial and a negative pregnancy test (urine) should be existent before the trial. Reliable contraception are systematic contraceptives (oral, implant, and injection) and diaphragm or condoms with spermicide. Women who are sterile by surgery or for more than two years postmenopausal can participate in the trial.

**The criteria for healthy volunteers include the following:**

- Male or female;
- Age ≥ 50 years.

*Exclusion* criteria

**Patients presenting with any of the following criteria will not be included in the trial:**

- Inability to obtain fluorescein angiography;
- Ophthalmic surgery or laser < 3 months before enrolment in one or both eyes;
- Any history of intravitreal steroids in one or both eyes;
- Systemic and/or intravitreal anti-VEGF treatment < 3 months before enrolment in one or both eyes;
- Patients with hypersensitivity against ranibizumab;
- Ocular inflammation (including trace or above) or external ocular inflammation in the study eye;
- Inability to give informed consent to participate in the study;
- Pregnancy and lactation, including women who are of childbearing age and not using medically acceptable effective contraception;
- Medical or psychological condition that would not permit the completion of the trial or signing of informed consent (legal representative accepted);
- Participation in other clinical trials including an investigational drug or device during the present clinical trial or within the last 4 weeks.

**The criteria for healthy volunteers include the following:**

- Ophthalmic surgery or laser < 3 months before enrolment;
- Relevant eye diseases except age-related cataract in one or both eyes;
- Inability to give informed consent to participate in the study;
- Pregnancy and lactation, including women who are of childbearing age and not using medically acceptable effective contraception;
- Medical or psychological condition that would not permit the completion of the trial or signing of informed consent (legal representative accepted);
- Participation in other clinical trials including an investigational drug or device during the present clinical trial or within the last 4 weeks.

**Table A1:** Trial Schedule.

	Visit	Visit 1 (Baseline)	Visit 2	Visit 3	Visit 4	Visit 5	Visit 6	Visit 7
Action								
Trial day		1	28 + 7 *	56 + 7 *	84 + 7 *	112 + 7 *	140 + 7 *	168 + 7 *
Trial week		0	4	8	12	16	20	24
Patient information and informed consent		X						
Medical history		X						
Demographics (e.g., sex, age and race)		X						
Inclusion/exclusion criteria		X						
Subjects with AMD								
Refraction		X	X	X	X	X	X	X
Best-corrected visual acuity (ETDRS chart)		X	X	X	X	X	X	X
Slit-lamp examination		X	X	X	X	X	X	X
Goldmann Applanation Tonometry		X	X	X	X	X	X	X
Indirect Ophthalmoscopy		X	X	X	X	X	X	X
Optical coherence tomography (OCT)		X	X	X	X	X	X	X
Blood sample collection		X	X	X	X	X	X	X
Urine Pregnancy Test (if applicable)		X						
Adverse events (AEs)		X	X	X	X	X	X	X
Study Medication/Injection		X	X	X	PRN	PRN	PRN	PRN
Goldmann Applanation Tonometry **		X	X	X	(X)	(X)	(X)	(X)
Safety call (1–2 days after injection)		X	X	X	(X)	(X)	(X)	(X)
End of trial (final visit)								X
Healthy volunteers								
Blood sample collection		X						
End of trial		X						

* The interval between intravitreal injections must be at least 28 days. ** 15–60 min after injection. X = study procedure was performed at that time.

**Table A2:** List of Analysed Antigens.

Antigen (Abbreviation)	Protein Name
MBP	Myelin basic protein
ACTA1	Actin, alpha skeletal muscle
SERPINA1	Alpha-1-antitrypsin
IGLL1	Immunoglobulin lambda-like polypeptide 1
ALB	Serum albumin
EIF4A1	Eukaryotic initiation factor 4A-I
MAPK3	Mitogen-activated protein kinase 3
HSPD1	60 kDa heat shock protein, mitochondrial
CKB	Creatine kinase B-type
SFN	14-3-3 protein sigma
GLUL	Glutamine synthetase
PEBP1	Phosphatidylethanolamine-binding protein 1
PKC	Protein kinase C
FN1	Fibronectin
USP10	Ubiquitin carboxyl-terminal hydrolase 10
TG	Thyroglobulin
groEL2	60 kDa chaperonin 2
PRKCSH	Glucosidase 2 subunit beta
TNNI3	Troponin I, cardiac muscle
HARS	Histidine--tRNA ligase, cytoplasmic
PPIA	Peptidyl-prolyl cis-trans isomerase A
Dermcidin	Dermcidin
TF	Serotransferrin
TTR	Transthyretin
ANXA5	Annexin A5
SPTA1	Spectrin alpha chain, erythrocytic 1
LYZ	Lysozyme C
CLUS	Clusterin
LPPR3	Phospholipid phosphatase-related protein type 3
DPYSL2	Dihydropyrimidinase-related protein 2
NTF3	Neurotrophin-3
CA2	Carbonic anhydrase 2
OGFR	Opioid growth factor receptor
Mucin 5B	Mucin-5B
BDNF	Brain-derived neurotrophic factor
HSPA1A	Heat shock 70 kDa protein 1A
SOD	Super oxid dismutase
INS	Insulin
GAPDH	Glyceraldehyde-3-phosphate dehydrogenase
CALR	Calreticulin
GNB1	Guanine nucleotide-binding protein G(I)/G(S)/G(T) subunit beta-1
UCHL1	Ubiquitin carboxyl-terminal hydrolase isozyme L1
TOP1	DNA topoisomerase 1
PDIA3	Protein disulfide-isomerase A3
VEGF	Vascular endothelial growth factor
GST	Glutathione S-transferase
SNCG	Gamma-synuclein
B-L-CRYS	Beta-L-Crystallin
TNF	Tumor necrosis factor
APOA1	Apolipoprotein A-I
SCFD1	Sec1 family domain-containing protein 1
ACO2	Aconitate hydratase, mitochondrial
HSPE1	10 kDa heat shock protein, mitochondrial
NTF4	Neurotrophin-4
GFAP	Glial fibrillary acidic protein
ENO2	Gamma-enolase
GPX4	Phospholipid hydroperoxide glutathione peroxidase, mitochondrial
SNCA	Alpha-synuclein
